# Prevention and treatment of social anxiety disorder in adolescents: mixed method randomised controlled trial of the guided online intervention SOPHIE

**DOI:** 10.1038/s41598-025-10193-w

**Published:** 2025-07-11

**Authors:** Noemi Walder, Thomas Berger, Dominique Hürzeler, Emily McDougal, Julian Edbrook-Childs, Stefanie J. Schmidt

**Affiliations:** 1 Division of Clinical Child and Adolescent Psychology, University of Bern, Bern, Switzerland; 2 Division of Clinical Psychology and Psychotherapy, University of Bern, Bern, Switzerland; 3 Anna Freud, London, UK; 4 University College London, London, UK

**Keywords:** Social anxiety disorder, Adolescents, Online intervention, Prevention, Internet-based, Psychology, Human behaviour

## Abstract

Social anxiety symptoms are highly prevalent in adolescents and negatively impact their social and academic functioning, highlighting the need for effective low-threshold interventions. This randomised controlled trial evaluated the guided online intervention SOPHIE for adolescents (*N* = 133; 11–17 years) with social anxiety disorder (SAD; treatment) or subclinical social anxiety (indicated prevention) compared to care-as-usual control condition and qualitatively explored their experiences. Outcomes were assessed at baseline, mid-intervention (4 weeks), post-intervention (8 weeks) and 5-month follow-up and analysed using linear mixed-effects models. SOPHIE did not significantly reduce social anxiety symptoms post-intervention but showed a significant between-group effect at follow-up (*d* = 0.67, 95%CI [0.32;1.02]). Subgroup analyses by diagnostic condition showed a significant between-group effect at follow-up in the subclinical social anxiety (*d* = 1.53, 95%CI [1.74;0.41]) but not in the SAD condition. Social functioning significantly improved at post-intervention and follow-up, with medium to large effects (post: *d*=-0.73, 95%CI [-1.08; -0.37]; follow-up: *d=*-0.32, 95%CI [-0.66; 0.02]). Qualitative interviews post-intervention revealed that participants found the intervention beneficial, although some found exposure exercises challenging and desired additional support. Very heterogeneous needs emerged regarding the guidance provided during the programme. Low-threshold online interventions for adolescents with social anxiety may be effective, particularly as an indicated prevention approach.

**Supplementary Information:**

The online version contains supplementary material available at 10.1038/s41598-025-10193-w.

## Introduction

Adolescence represents an important period in the development of social anxiety disorder (SAD), with a mean age of onset of 14 years^1^. In population-based adolescent samples, the point prevalence of clinician-assessed SAD-criteria is 2.6%, while up to 50% report subclinical levels of social anxiety symptoms^2–7^. If left untreated, SAD has a high likelihood of persisting into adulthood and may lead to the development of other disorders as well as lower social and academic functioning^8–12^. Highly scalable, low-threshold intervention programs, such as online interventions, could be a promising strategy to prevent such negative long-term consequences and to address the care gap that is especially pronounced in adolescents^13^.

Online interventions have shown small but significant effects in reducing anxiety symptoms in adolescents with an anxiety disorder^14–16^ but research on online delivered prevention is still inconclusive. In face-to-face settings, meta-analytic evidence has shown that indicated transdiagnostic interventions yield small but significant effects in reducing anxiety symptoms among adolescents at risk for several anxiety disorders^17^. In online settings however, even though some studies found a small significant reduction in subclinical anxiety symptoms, meta-analytic results on universal and targeted prevention programmes could not confirm this^18,19^. Low participation and high dropout rates and challenges regarding the implementation and sustaining use of online interventions in adolescent studies may contribute to these low effects. Human support in the form of guidance may help to address these issues^20,21^ as guided interventions based on Cognitive Behavioural Therapy (CBT) have often demonstrated to be more effective in adolescent samples than unguided self-help or other psychological interventions^22^.

Next to human guidance, tailoring interventions specifically to SAD may yield better outcomes as adolescents with SAD symptoms benefited more from SAD-specific content than from interventions focusing on anxiety in general^23–28^. Such interventions specifically designed for SAD could target mechanisms involved in developing and maintaining SAD. Etiological models of social anxiety^29–32^ propose overlapping factors that contribute to maintaining SAD and form the basis of SAD-specific interventions. These include cognitive processes such as negative social-evaluative cognitions and self-focus during social situations, as well as behavioural processes such as avoidance behaviours beforehand and safety behaviours during social interactions^33^. Randomised controlled trials (RCT) investigating online interventions based on such models, specifically the Cognitive Model of Social Phobia by Clark and Wells and the Cognitive-Behavioural Model of Anxiety in Social Phobia by Rapee and Heimberg, in clinical adolescent samples with SAD showed significant reductions in social anxiety and improvements in social functioning^34–36^. None of these RCTs has specifically targeted subclinical levels of social anxiety.

Previous research on the evaluation of online interventions is mainly based on RCTs. Integrating qualitative elements in RCTs could offer a more comprehensive understanding of the intervention’s impact and the contextual factors influencing its efficacy. Further, it offers the possibility to involve adolescents’ voices thereby democratising inputs to research^37,38^. A previous RCT included a qualitative evaluation of adolescents’ experiences with an online intervention for SAD: Adolescents appreciated the autonomy when working through the modules at their own pace and the flexibility in contact with the person providing guidance^39^. They considered the exposure exercises to be the most challenging but also the most helpful ones. These qualitative results were reported separately from the quantitative efficacy results^36,39^ although a combined discussion could provide a more integrated understanding of the effects^40^.

Overall, initial studies suggest positive effects of online interventions on adolescents with SAD, but evidence on those with subclinical symptoms is limited. There is little qualitative research on adolescents’ experiences of using such interventions. Thus, the primary aim of this RCT was to evaluate the efficacy of an online intervention called SOPHIE^41^ developed to reduce subclinical social anxiety (i.e., indicated prevention) and SAD (i.e., intervention) by targeting the psychological mechanisms postulated by the Clark & Wells’ Cognitive Model^30^ (i.e., self-focused attention, negative automatic thoughts during a social event, and pre- and post-event processing of this situation, as well as safety behaviour and avoidance) by comparing the effects of the intervention group with those of the care-as-usual (CAU) control group on social anxiety symptoms at post- (primary outcome; 2 months after randomisation) and follow-up (5 months after randomisation) assessments. Secondary outcomes included remission of SAD diagnosis, generalised anxiety, depression, self-esteem, quality of life, level of functioning, and social anxiety assessed by guardians. Further, the participants’ experiences of using the SOPHIE program were explored through qualitative interviews.

## Results

### Participants

**Fig. 1:**
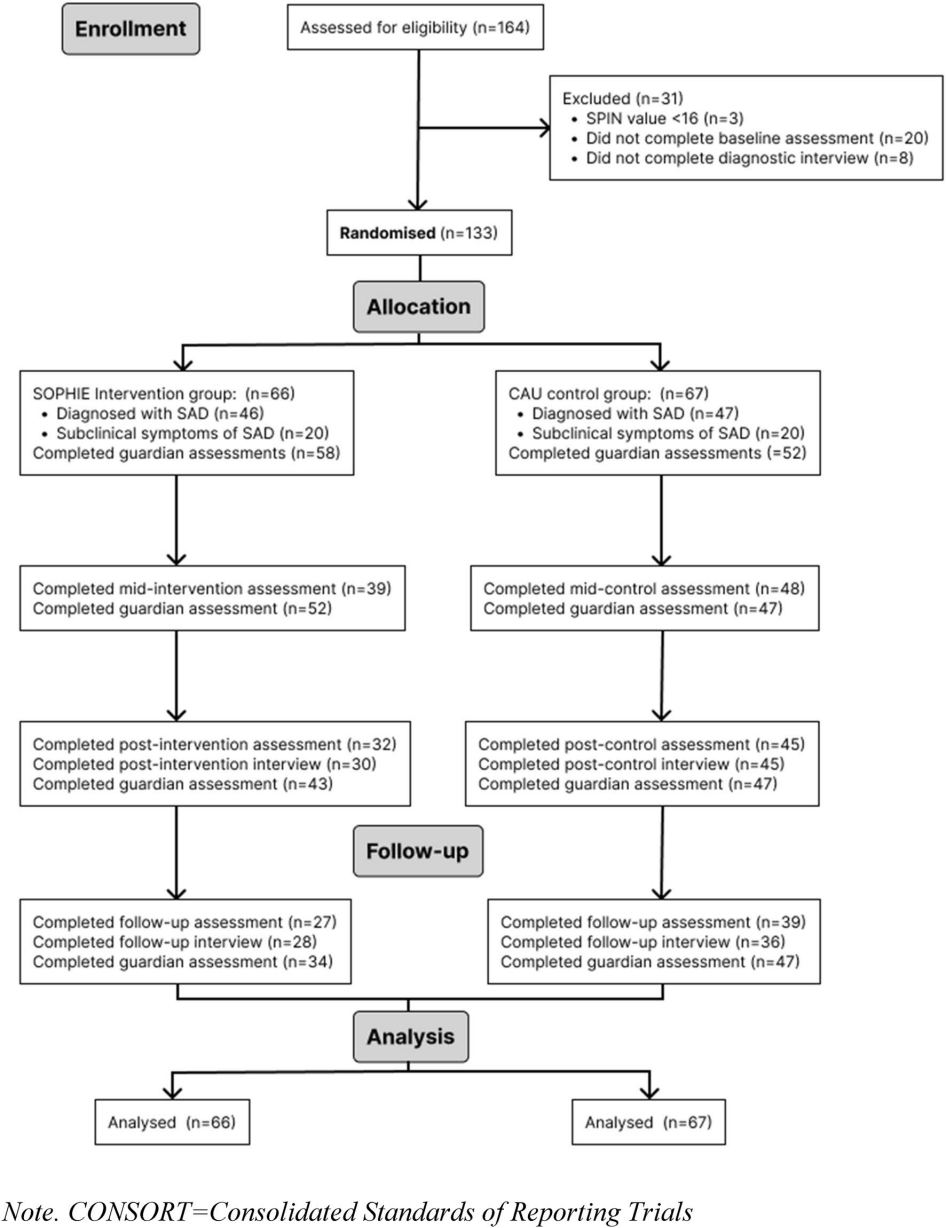
CONSORT diagram of the SOPHIE study. Mid-intervention assessment (4 weeks), post-intervention assessment (8 weeks) and follow-up assessment (5 months after randomisation).

Recruitment of adolescents aged 11 to 17 years started in August 2021 and ended in August 2023 due to regulatory reasons. Last assessment was in March 2024. Recruitment of participants with subclinical social anxiety resulted in a lower sample size than targeted even though a comprehensive recruitment plan was followed. The SAD-group sample size was increased from the planed sample size of *N* = 56 to *N* = 80 participants, to account for dropouts and incomplete assessments^42^. In total, 164 participants provided informed consent or assent. Of these, 133 completed baseline assessments and were randomised to the SOPHIE intervention or CAU group. Additionally, 117 guardians completed assessments at least once. The CONSORT flow diagram (see Fig. 1) illustrates the progression of participants while baseline characteristics are presented in Table 1. There were no significant differences between study groups in terms of demographic, clinical, or outcome variables.

**Table 1:** Demographic and diagnostic characteristics of the adolescent sample (*N* = 133) and their guardians (*N* = 117).

	SOPHIE	CAU	Statistics
**Demographic data**			
Gender (n, %)			*X*^*2*^(2) = 3.27, *p* = 0.20
*Female*	49 (74.2)	54 (80.6)	
*Male*	14 (21.2)	13 (19.4)	
*Diverse*	3 (4.6)	0 (0.0)	
Age (M, SD)	14.71 (16.1)	14.99 (1.61)	*t*(130.97) = 1.01, *p* = 0.32
**Living situation (n**,** %)**			*X*^*2*^(3) = 1.33, *p* = 0.72
*Parental Home*	62 (93.9)	61 (91.0)	
*Boarding School/Youth home*	1 (1.5)	2 (3.0)	
*Own/Shared Flat*	0 (0.0)	1 (1.5)	
*Other*	3 (4.6)	3 (4.5)	
Number of persons per household (adolescent included) (n, %)			*X*^*2*^(4) = 0.58, *p* = 0.97
*2*	6 (9.1)	7 (10.5)	
*3*	27 (40.9)	26 (38.8)	
*4*	20 (30.3)	22 (32.8)	
*5*	11 (16.7)	9 (13.4)	
*> 5*	2 (3.0)	3 (4.5)	
Number of siblings (M, SD)	1.18 (0.93)	1.32 (0.99)	*t*(129.35) = 0.82, *p* = 0.42
Socioeconomic status (Family Affluence Scale, M, SD)	9.82 (1.59)	9.48 (1.95)	*t*(126.31) = −1.09, *p* = 0.28
**Level of functioning (M**,** SD)**			
*Current social functioning*	6.64 (1.27)	6.39 (1.26)	*t*(130.89) = −1.13, *p* = 0.26
*Past social functioning*	6.76 (1.25)	6.46 (1.19)	*t*(130.34) = −1.39, *p* = 0.17
*Current role functioning*	6.91 (1.86)	7.12 (1.72)	*t*(129.83) = 0.68, *p* = 0.50
*Past role functioning*	7.03 (1.75)	7.25 (1.40)	*t*(123.93) = 0.81, *p* = 0.42
**Diagnostics**			
Primary Diagnosis (n, %):			*X*^*2*^(13) = 13.21, *p* = 0.43
Social anxiety disorder (SAD)	41 (62.2)	47 (70.1)	
Other anxiety disorder			
*Agoraphobia*	0 (0.0)	1 (1.5)	
*GAD*	0 (0.0)	1 (1.5)	
*Panic disorder*	0 (0.0)	1 (1.5)	
*Separation anxiety disorder*	2 (3.0)	0 (0.0)	
*Selective Mutism*	0 (0.0)	1 (1.5)	
Mood disorder	5 (7.6)	1 (1.5)	
ADHD	1 (1.5)	0 (0.0)	
Anorexia nervosa	1(1.5)	0 (0.0)	
No primary diagnosis (subclinical only)	16 (24.2)	15 (22.4)	
More than one current disorder	18	20	*X*^*2*^(1) = 0.02, *p* = 0.89
Past disorder(s)	14	15	*X*^*2*^(1) < 0.01, *p* = 1.00
**Parents/Guardians**	*N* = 61	*N* = 56	
Gender (n, %)			*X*^*2*^(2) = 0.95, *p* = 0.62
*Female*	52 (85.3)	48 (85.7)	
*Male*	8 (13.1)	8 (14.3)	
*Diverse*	1 (1.6)	0 (0.0)	
Age (M, SD)	46.71 (5.42)	47.76 (5.25)	*t*(113.4) = 1.07, *p* = 0.29
Highest level of education based on ISCED-2011-Level (n, *%*)			*X*^*2*^(7) = 8.77, *p* = 0.27
*Primary education*	2 (3.3)	1 (1.8)	
*Lower secondary education*	5 (8.2)	5 (8.9)	
*Upper secondary education*	22 (36.1)	15 (26.9)	
*Bachelor or equivalent*	12 (19.7)	12 (21.4)	
*Master or equivalent*	17 (27.8)	19 (33.9)	
*Doctorate or equivalent*	3 (4.9)	4 (7.1)	
Employment (n, %)			*X*^*2*^(5) = 2.38, *p* = 0.79
*Fulltime*	24 (39.3)	18 (32.1)	
*Part time*	30 (49.2)	32 (57.1)	
*Unemployed*	3 (4.9)	2 (3.6)	
*In education/student*	0 (0.0)	2 (3.6)	
*Housewife/househusband*	4 (6.6)	2 (3.6)	
Relationship status (n, %)			*X*^*2*^(6) = 5.62, *p* = 0.47
*Single*	1 (1.7)	3 (5.4)	
*In relationship*	5 (8.2)	4 (7.1)	
*Married*	45 (73.7)	45 (80.4)	
*Separated*	3 (4.9)	0 (0.0)	
*Divorced*	7 (11.5)	4 (7.1)	

Notes. M = mean; SD = standard deviation; SAD = social anxiety disorder; GAD; generalised anxiety disorder; ADHD; attention deficit hyperactivity disorder.

During the intervention period, *n* = 18 (intervention group: *n* = 9; CAU group *n* = 9) adolescents received weekly or biweekly psychotherapy, *n* = 5 (intervention group: *n* = 5; CAU group *n* = 0) adolescents were supported by psychosocial professionals (e.g., social workers), and *n* = 5 (intervention group: *n* = 3; CAU group *n* = 2) adolescents were medicated with psychotropic drugs. For the intervention group, Table 2 provides details on adherence rates, amount of guidance, any reported negative effects due to the intervention and participants’ overall satisfaction with the intervention.

**Table 2:** Adherence to the intervention, guidance, negative effects due to and satisfaction with the intervention for the intervention group (*n* = 66).

	M(SD)
Adherence	
*Time spent in minutes*	146.12 (170.11)
*Number of days logged in*	10.95 (10.88)
*Number of exercises practiced*	25.83 (38.01)
*Number of modules accessed*	4.39 (2.68)
*Module 1 accessed*,* n (%)*	64 (97)
*Module 2 accessed n (%)*	51 (77)
*Module 3 accessed n (%)*	39 (59)
*Module 4 accessed n (%)*	34 (51)
*Module 5 accessed n (%)*	28 (42)
*Module 6 accessed n (%)*	27 (41)
*Module 7 accessed n (%)*	20 (30)
*Module 8 accessed n (%)*	20 (30)
Guidance per participant (in minutes)	89.21 (52.26)
Satisfaction with the intervention	*M* = 3.24, *SD* = 0.53
Number of negative effects	*M* = 0.33, *SD* = 1.14
Reported negative effects:	Reported by *n* participants
Weekly support in the intervention was perceived as disruptive	3
Relationship with family worsened after completion of intervention	2
Feeling worse after intervention completion	1
Suffering more from past events	1
Less time spent on hobbies and social activities while using the intervention	1
Says of thinking learned in the intervention were perceived as harmful	1
Feelings of loneliness have increased during use of the intervention	1
Reduced motivation to start psychotherapy	1

Notes: Satisfaction with the intervention and number of negative effects are reported for adolescents in the intervention group who completed post-assessment (*n* = 33). Of these adolescents, 4 (12%) reported one or more negative effects. One adolescent reported 6 negative effects, 2 adolescents each reported 2 negative effects, 1 adolescent reported 1 negative effects.

### Missingness and dropout analysis

The percentage of missing values on the primary outcome (SPIN) was 27% and on secondary outcomes 28% in total. Telephone interviews had higher missingness (44% at post-assessment and 53% at follow-up). Demographic variables and baseline assessments did not predict missingness at mid-, post-, or follow-up assessments supporting missingness at random (MAR). Only younger age at baseline was significantly associated with missingness at follow-up (*β* = 0.27, *p* < 0.05*)*. Adolescents in the CAU control group tended to complete more assessments, but the difference was only significant at post-assessment (*X*^*2*^_(1)_ = 6.04, *p* < 0.05*)*.

### Efficacy analysis

**Fig. 2:**
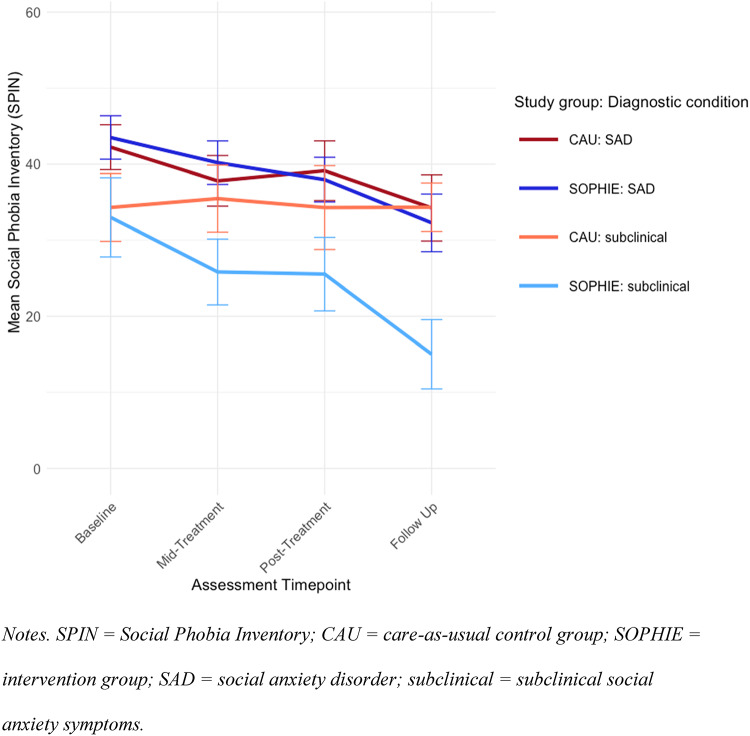
Observed mean SPIN scores and their 95% confidence intervals at all assessment points divided by diagnostic condition and study group.

Mean SPIN scores over time are shown separately for both study groups (SOPHIE vs. CAU) and diagnostic conditions (SAD vs. subclinical) in Fig. 2. Observed and estimated means of primary and secondary outcomes of the linear mixed-effects models from baseline to post-intervention are reported in Table 3. Table 4 displays intercept and estimates of the linear mixed-effects models on primary and secondary outcomes for time and group interaction from baseline to post and corresponding between and within group effect sizes. Observed and estimated means at follow-up are reported in Table 5, and coefficients derived of the linear mixed-effects models and effect sizes are displayed in Table 6.

**Table 3:** Observed and estimated means for primary and secondary outcomes from baseline to post-intervention assessment.

Measure	Condition	Baseline (observed)		Baseline (estimated)		Mid-treatment (observed)		Mid-treatment (estimated)		Post (observed)		Post (estimated)	
		Mean (SD)	n	Mean (SE)	n	Mean (SD)	n	Mean (SE)	n	Mean (SD)	n	Mean (SE)	n
SPIN	SOPHIE	40.32 (11.50)	66	39.93 (0.91)	66	36.43 (11.68)	42	35.98 (1.13)	66	33.60 (11.95)	37	34.82 (1.20)	66
	CAU	39.87 (10.84)	67	39.85 (85)	67	37.10 (11.14)	50	37.12 (1.03)	67	37.83 (13.49)	52	37.97 (1.01)	67
*SPIN*	*SOPHIE SAD*	*43.50 (9.87)*	*46*	*43.16 (0.99)*	*46*	*40.19 (9.90)*	*31*	*39.62 (1.08)*	*46*	*37.96 (10.18)*	*24*	*36.60 (1.35)*	*46*
	*CAU SAD*	*42.23 (10.32)*	*47*	*43.04 (0.98)*	*47*	*37.80 (11.64)*	*35*	*38.50 (1.13)*	*47*	*39.13 (13.73)*	*38*	*39.37 (1.08)*	*47*
*SPIN*	*SOPHIE subclinical*	*33.00 (11.89)*	*20*	*32.32 (1.89)*	*20*	*25.82 (9.87)*	*11*	*26.74 (2.52)*	*20*	*25.54 (11.03)*	*13*	*29.46 (2.35)*	*20*
	*CAU subclinical*	*34.30 (10.18)*	*20*	*32.67 (1.90)*	*20*	*35.47 (10.06)*	*15*	*34.18 (2.17)*	*20*	*34.29 (12.85)*	*14*	*35.13 (2.34)*	*20*
PHQ-9	SOPHIE	12.09 (5.95)	66	11.40 (0.39)	66	9.36 (5.39)	42	8.52 (0.49)	66	7.41 (4.95)	37	6.88 (0.52)	66
	CAU	10.66 (5.27)	67	11.10 (0.39)	67	9.16 (4.77)	50	9.92 (0.45)	67	9.49 (5.10)	49	10.14 (0.46)	67
GAD-7	SOPHIE	11.20 (4.65)	66	10.67 (0.35)	66	8.83 (4.56)	41	8.56 (0.44)	66	7.00 (4.58)	37	6.93 (0.46)	66
	CAU	10.45 (4.24)	67	10.53 (0.34)	67	8.71 (4.30)	49	9.14 (0.40)	67	9.42 (4.89)	52	9.85 (0.39)	67
SAS-A	SOPHIE	67.86 (11.51)	66	68.96 (0.92)	66	65.05 (10.38)	40	65.55 (1.18)	66	57.95 (14.35)	37	60.16 (1.23)	66
	CAU	70.02 (10.71)	67	69.30 (0.92)	67	68.69 (10.87)	49	67.41 (1.07)	67	67.25 (11.77)	52	66.07 (1.04)	67
KIDSSCREEN	SOPHIE	35.32 (3.71)	66	35.88 (0.39)	66	36.08 (4.47)	40	36.30 (0.49)	66	38.35 (4.81)	37	38.43 (0.51)	66
	CAU	36.42 (3.56)	67	36.10 (0.39)	67	36.38 (4.60)	49	36.05 (0.45)	67	36.16 (4.34)	52	35.83 (0.44)	67
RSES	SOPHIE	15.79 (2.08)	66	15.79 (0.25)	66	15.93 (2.52)	40	16.09 (0.32)	66	16.19 (2.79)	37	16.09 (0.33)	66
	CAU	15.87 (2.32)	67	15.82 (0.24)	67	15.54 (2.34)	48	15.67 (0.29)	67	15.42 (2.71)	52	15.31 (0.28)	67
GF social*	SOPHIE	6.64 (1.27)	66	6.46 (0.06)	66	N/A		N/A		6.70 (1.40)	45	6.71 (0.06)	66
	CAU	6.39 (1.26)	67	6.49 (0.09)	67	N/A		N/A		6.29 (1.24)	29	6.22 (0.10)	67
GF role*	SOPHIE	6.91 (1.86)	66	7.23 (0.04)	66	N/A		N/A		6.83 (1.47)	45	7.09 (0.05)	66
	CAU	7.12 (1.72)	67	7.19 (0.08)	67	N/A		N/A		7.29 (1.38)	29	7.12 (0.10)	67
ESAK	SOPHIE	40.92 (9.23)	61	41.20 (0.66)	61	40.20 (9.83)	54	41.05 (0.70)	61	40.30 (11.37)	44	41.13 (0.77)	61
	CAU	41.04 (9.33)	55	41.20 (0.70)	55	40.55 (10.31)	49	39.60 (0.74)	55	41.27 (11.49)	48	39.94 (0.75)	55

Notes. All linear mixed-effects models included the baseline value of investigated outcome value as a fixed covariate and a random effect of participants. SPIN = Social Phobia Inventory; SAD = diagnostic group including adolescents with a social anxiety disorder at baseline; subclinical = diagnostic group including adolescents with subclinical levels of social anxiety at baseline; PHQ-9 = 9-item version of the Patient Health Questionnaire; GAD-7: 7-item version of the Generalized Anxiety Disorder; SAS-A = Social Anxiety Scale for Adolescents; KIDSSCREEN = quality of life assessment KIDSCREEN-10; RSES = Rosenberg Self-esteem Scale; GF social = Global functioning subscale social; GF role = Global functioning subscale role; ESAK = Parents’ Questionnaire on Social Anxieties in Childhood and Adolescence.

* Estimated means and standard errors for GF social and GF role are from the follow-up mixed model.

**Table 4:** Results from the linear mixed-effects models from baseline to post and between- and within-group effect sizes displayed in cohen’s *d*.

		Coefficients from baseline to post linear mixed-effects models (SOPHIE vs. CAU)			Effect sizes Cohen’s d [95% CI]		
		*Estimates* (SE)	t [CI]	p	Between-group	Within-group baseline post	
						SOPHIE	CAU
SPIN	Intercept	6.90 (1.98)	3.48 [3.00;10.79]	**0.001**			
	Mid-intervention	−1.22 (1.78)	−0.69 [−4.72; 2.28]	0.492	0.13 [−0.21; 0.47]		
	Post-intervention	−3.23 (1.81)	−1.78 [−6.79; 0.34]	0.076	0.35 [0.01; 0.69]	0.60 [0.25; 0.95]	0.24 [−0.10; 0.58]
*SPIN SAD*	*Intercept*	*4.17 (2.49)*	*1.68 [−0.73; 9.07]*	*0.095*			
	*Mid-intervention*	*0.99 (1.93)*	*0.52 [−2.80; 4.79]*	*0.607*	*−0.12 [−0.46; 0.22]*		
	*Post-intervention*	*−2.90 (2.00)*	*−1.45 [−6.84; 1.04]*	*0.148*	*0.28 [−0.06; 0.62]*	*0.68 [0.34; 1.04]*	*0.44 [0.10; 0.78]*
*SPIN*	*Intercept*	*9.47 (3.94)*	*2.40 [1.63; 17.31]*	***0.019***			
*(subclinical)*	*Mid-intervention*	*−7.08 (3.80)*	*−1.86 [−14.63; 0.47]*	*0.066*	*0.39 [0.73; 0.05]*		
	*Post-intervention*	*−5.31 (3.72)*	*−1.43 [−12.71; 2.09]*	*0.157*	*0.31 [−0.04; 0.65]*	*0.16 [−0.18; 0.51]*	*−0.15 [−0.49; 0.19]*
PHQ-9	Intercept	2.25 (0.56)	4.03 [1.15; 3.35]	**< 0.001**			
	Mid-intervention	−1.70 (0.80)	−2.14 [−3.27; −0.14]	**0.033**	0.02 [−0.32; 0.36]		
	Post-intervention	−3.56 (0.82)	−4.35 [−5.18; −1.95]	**< 0.001**	0.38 [0.04; 0.72]	0.82 [0.46; 1.17]	0.18 [−0.15; 0.53]
GAD-7	Intercept	1.96 (0.54)	3.59 [0.88; 3.03]	**< 0.001**			
	Mid-intervention	−0.73 (0.72)	−1.01 [−2.14; 0.69]	0.315	0.17 [−0.17; 0.51]		
	Post-intervention	−3.05 (0.73)	−4.20 [−4.49; −1.62]	**< 0.001**	0.85 [0.49; 1.20]	1.14 [0.77; 1.50]	0.23 [−0.11; 0.58]
SAS-A	Intercept	11.03 (3.29)	3.35 [4.56; 17.50]	**0.001**			
	Mid-intervention	−1.53 (1.92)	−0.80 [−5.30; 2.25]	0.427	0.21 [−0.14–0.55]		
	Post-intervention	−5.57 (1.93)	−2.89 [−9.37; −1.77]	**0.004**	0.64 [0.29–0.99]	1.00 [0.64; 1.36]	0.41 [0.06; 0.74]
KIDSSCREEN	Intercept	7.48 (2.16)	3.46 [3.23; 11.74]	**0.001**			
	Mid-intervention	0.47 (0.79)	0.79 [−1.08; 2.02]	0.551	−0.06 [−0.40; 0.28]		
	Post-intervention	2.82 (0.79)	3.56 [1.26; 4.38]	**< 0.001**	−0.68 [−1.02; −0.33]	−0.70 [−1.05; −0.34]	0.08 [−0.26; 0.42]
RSES	Intercept	5.94 (0.87)	6.80 [4.22; 7.67]	**< 0.001**			
	Mid-intervention	0.46 (0.54)	0.85 [−0.60; 1.52]	0.398	−0.18 [−0.52; 0.16]		
	Post-intervention	0.81 (0.54)	1.50 [−0.25; 1.88]	0.134	−0.31 [−0.65; 0.03]	−0.13 [−0.47; 0.21]	0.23 [−0.11; 0.57]
ESAK	Intercept	−0.02 (1.79)	−0.01 [−3.54; 3.50]	0.991			
	Mid-intervention	1.45 (1.23)	1.18 [−0.96; 3.86]	0.237	−0.25 [−0.59; 0.09]		
	Post-intervention	1.18 (1.28)	0.93 [−1.33; 3.69]	0.355	−0.19 [−0.53; 0.15]	0.13 [−0.33; 0.35]	0.21 [−0.13; 0.55]

Notes: All linear mixed-effects models included the baseline value of investigated outcome value as a fixed covariate and a random effect of participants. SPIN = Social Phobia Inventory; PHQ-9 = 9-item version of the Patient Health Questionnaire; GAD-7: 7-item version of the Generalized Anxiety Disorder; SAS-A = Social Anxiety Scale for Adolescents; KIDSSCREEN = quality of life assessment KIDSCREEN-10; RSES = Rosenberg Self-esteem Scale; ESAK = Parents’ Questionnaire on Social Anxieties in Childhood and Adolescence.

**Table 5:** Observed and estimated means for primary and secondary outcomes from baseline to follow-up assessment.

Measure	Condition	Baseline (observed)		Baseline (estimated)		Follow-up (observed)		Follow-up (estimated)	
		Mean (SD)	n	Mean (SE)	n	Mean (SD)	n	Mean (SE)	n
SPIN	SOPHIE	40.32 (11.50)	66	39.80 (1.02)	66	36.43 (11.68)	42	28.11 (1.42)	66
	CAU	39.87 (10.84)	67	39.71 (1.01)	67	37.10 (11.14)	50	35.39 (1.25)	67
*SPIN*	*SOPHIE SAD*	*43.50 (9.87)*	*46*	*43.10 (1.14)*	*46*	*32.27 (13.12)*	*22*	*32.21 (1.59)*	*46*
	*CAU SAD*	*42.23 (10.32)*	*47*	*42.99 (1.13)*	*47*	*34.24 (15.24)*	*29*	*35.65 (1.39)*	*47*
*SPIN*	*SOPHIE subclinical*	*33.00 (11.89)*	*20*	*32.07 (1.94)*	*20*	*15.00 (10.42)*	*9*	*17.76 (2.79)*	*20*
	*CAU subclinical*	*34.30 (10.18)*	*20*	*32.52 (1.94)*	*20*	*34.33 (7.27)*	*12*	*35.20 (2.44)*	*20*
PHQ-9	SOPHIE	12.09 (5.95)	66	11.34 (0.42)	66	9.36 (5.39)	42	8.09 (0.60)	66
	CAU	10.66 (5.27)	67	10.99 (0.42)	67	9.16 (4.77)	50	8.57 (0.53)	67
GAD-7	SOPHIE	11.20 (4.65)	66	10.61 (0.38)	66	8.83 (4.56)	41	7.61 (0.54)	66
	CAU	10.45 (4.24)	67	10.47 (0.37)	67	8.71 (4.30)	49	8.49 (0.47)	67
SAS-A	SOPHIE	67.86 (11.51)	66	68.76 (1.08)	66	65.05 (10.38)	40	59.66 (1.52)	66
	CAU	70.02 (10.71)	67	69.16 (1.07)	67	68.69 (10.87)	49	63.22 (1.33)	67
KIDSSCREEN	SOPHIE	35.32 (3.71)	66	35.92 (0.42)	66	36.08 (4.47)	40	41.78 (0.60)	66
	CAU	36.42 (3.56)	67	36.13 (0.42)	67	36.38 (4.60)	49	40.91 (0.53)	67
RSES	SOPHIE	15.79 (2.08)	66	15.78 (0.25)	66	15.93 (2.52)	40	15.68 (0.37)	66
	CAU	15.87 (2.32)	67	15.82 (0.25)	67	15.54 (2.34)	48	15.33 (0.32)	67
GF social	SOPHIE	6.64 (1.27)	66	6.46 (0.06)	66	6.85 (1.56)	26	6.66 (0.06)	66
	CAU	6.39 (1.26)	67	6.49 (0.09)	67	6.42 (1.42)	27	6.43 (0.11)	67
GF role	SOPHIE	6.91 (1.86)	66	7.23 (0.04)	66	6.93 (1.64)	26	6.92 (0.05)	66
	CAU	7.12 (1.72)	67	7.19 (0.08)	67	7.31 (1.82)	27	7.07 (0.11)	67
ESAK	SOPHIE	40.92 (9.23)	61	41.12 (0.79)	61	40.20 (9.83)	54	37.64 (0.99)	66
	CAU	41.04 (9.33)	55	41.12 (0.83)	55	40.55 (10.31)	49	38.40 (0.89)	67

Notes. All linear mixed-effects models included the baseline value of investigated outcome value as a fixed covariate and a random effect of participants. SPIN = Social Phobia Inventory; SAD = diagnostic group including adolescents with a social anxiety disorder at baseline; subclinical = diagnostic group including adolescents with subclinical levels of social anxiety at baseline; PHQ-9 = 9-item version of the Patient Health Questionnaire; GAD-7: 7-item version of the Generalized Anxiety Disorder; SAS-A = Social Anxiety Scale for Adolescents; KIDSSCREEN = quality of life assessment KIDSCREEN-10; RSES = Rosenberg Self-esteem Scale; GF social: Global Functioning Scale, subscale social; GF role: Global Functioning Scale, subscale role; ESAK = Parents’ Questionnaire on Social Anxieties in Childhood and Adolescence.

**Table 6:** Results from the linear mixed-effects models from baseline to follow-up and between and within group effect sizes displayed in cohen’s *d*.

		Coefficients from baseline to follow-up linear mixed-effects models (SOPHIE vs. CAU)			Effect sizes Cohen’s d [95% CI]			
		Estimates (SE)	t [CI]	p	Between-group	Within-group baseline follow-up		
						SOPHIE	CAU	
SPIN	Intercept	7.48 (2.23)	3.36 [3.10; 11.85]	**0.001**				
	Follow-up	−7.37 (2.07)	−3.57 [−11.44; −3.31]	**< 0.001**	0.67 [0.32; 1.02]	1.17 [0.80; 1.54]	0.16 [−0.18; 0.50]	
*SPIN*	*Intercept*	*3.64 (2.85)*	*1.28 [−1.98; 9.25]*	*0.203*				
*SAD*	*Follow-up*	*−3.55 (2.34)*	*−1.52 [−8.15; 1.06]*	*0.130*	*1.21[0.77; 1.65]*	*1.17 [0.73; 1.62]*	*0.85 [0.43; 1.28]*	
*SPIN*	*Intercept*	*11.75 (4.04)*	*2.91 [3.74; 19.76]*	***0.004***				
*subclinical*	*Follow-up*	*−17.00 (4.04)*	*−4.21 [−25.00; −8.99]*	***< 0.001***	*1.53 [0.82; 2.23]*	*1.37 [0.68; 2.05]*	*−0.28 [−0.90; 0.35]*	
PHQ-9	Intercept	2.55 (0.60)	4.28 [1.38; 3.73]	**< 0.001**				
	Follow-up	−0.82 (0.91)	−0.90 [−2.61; 0.96]	0.366	0.10 [−0.24; 0.44]	0.77 [0.42; 1.13]	0.63 [0.28; 0.97]	
GAD-7	Intercept	2.03 (0.59)	3.42 [0.86; 3.19]	**0.001**				
	Follow-up	−1.02 (0.81)	−1.25 [−2.62; 0.58]	0.212	0.21 [−0.13; 0.55]	0.80 [0.44; 1.15]	0.57 [0.23; 0.92]	
SAS-A	Intercept	13.00 (3.87)	3.36 [5.39; 20.62]	**0.001**				
	Follow-up	−3.17 (2.24)	−1.42 [−7.57; 1.23]	0.158	0.31 [−0.03; 0.65]	0.86 [0.50; 1.21]	0.61 [0.26; 0.95]	
KIDSCREEN	Intercept	6.87 (2.24)	3.06 [2.46; 11.28]	**0.002**				
	Follow-up	1.09 (0.92)	1.18 [−0.72; 2.89]	0.239	−0.19 [−0.53; 0.15]	−1.40 [−1.78; −1.01]	−1.34 [−1.61; −0.86]	
RSES	Intercept	7.00 (0.87)	8.02 [5.29; 8.72]	**< 0.001**				
	Follow-up	0.38 (0.58)	0.66 [−0.76; 1.53]	0.511	−0.13 [−0.47; 0.22]	0.20 [−0.15; 0.54]	0.21 [−0.13; 0.55]	
GF social	Intercept	0.62 (0.25)	2.51 [0.14; 1.11]	**0.012**				
	Post-intervention	0.51 (0.11)	4.83 [0.31; 0.72]	**< 0.001**	−0.73 [−1.08; −0.37]	−0.52 [−0.87; −0.18]	0.35 [0.01; 0.70]	
	Follow-up	0.26 (0.12)	2.23 [0.03; 0.49]	**< 0.001**	−0.32 [−0.66; 0.02]	−0.41 [−0.76; −0.07]	0.08 [−0.26; 0.42]	
GF role	Intercept	0.42 (0.16)	2.55 [0.10; 0.74]	**0.011**				
	Post-intervention	−0.08 (0.11)	−0.72 [−0.29; 0.14]	0.470	0.06 [−0.28; 0.40]	0.40 [0.06; 0.75]	0.09 [−0.25; 0.43]	
	Follow-up	−0.20 (0.12)	−1.66 [0.43; 0.04]	0.098	0.23 [−0.11; 0.57]	0.87 [0.51; 1.22]	0.15 [−0.19; 0.49]	
ESAK	Intercept	−0.13 (2.10)	−0.06 [−4.27; 4.00]	0.949				
	Follow-up	−0.76 (1.51)	−0.50 [−3.72; 2.21]	0.616	0.10 [−0.24; 0.44]	0.48 [0.14; 0.83]	0.39 [0.05; 0.73]	

Notes. All linear mixed-effects models included the baseline value of investigated outcome value as a fixed covariate and a random effect of participants. SPIN = Social Phobia Inventory; PHQ-9 = 9-item version of the Patient Health Questionnaire; GAD-7: 7-item version of the Generalized Anxiety Disorder; SAS-A = Social Anxiety Scale for Adolescents; KIDSSCREEN = quality of life assessment KIDSCREEN-10; RSES = Rosenberg Self-esteem Scale; GF social: Global Functioning Scale, subscale social; GF role: Global Functioning Scale, subscale role; ESAK = Parents’ Questionnaire on Social Anxieties in Childhood and Adolescence.

The primary outcome (SPIN score at post-assessment) did not differ significantly between the two study groups. However, the SOPHIE group had significantly lower SPIN scores compared to the CAU group at follow-up (*p* < 0.001; Table 6) with a medium to large between-group effect size. Subgroup analyses were run for both diagnostic condition SAD and subclinical social anxiety. For the SAD condition, there was no significant difference between the intervention group and the control group at post- and follow-up assessment. For the subclinical condition, no significant difference was found at post-assessment, however, the intervention group revealed significantly lower levels of social anxiety compared to the control group at follow-up (*p* < 0.001; Table 6) with a large between-group effect size.

The secondary outcomes at post-assessment including social functioning, depression, general anxiety, social fears and avoidance, and quality of life improved significantly in the intervention group compared to CAU (*p* < 0.004; Table 4) with small to medium between-group effect sizes but not role functioning, self-esteem and guardian rated social anxiety (*p ≥* 0.134; Table 4). At follow-up, significant improvements could be maintained in social functioning (*p* < 0.001; Table 6) with a small between-group effect, while role functioning, depression, general anxiety, social fears and avoidance, quality of life, self-esteem and guardian rated social anxiety did not change significantly (*p ≥* 0.098; Table 6).

Remission of SAD in the clinical group yielded no significant differences between the intervention and control condition at post- and follow-up assessment (*p ≥* 0.118). In the clinical intervention group, 63% of the adolescents met diagnostic criteria for SAD at post-assessment and 80% at follow-up; in the clinical CAU group, 74% at post-assessment and 65% at follow-up.

The sensitivity analyses using the per-protocol sample (i.e., participants who completed baseline, mid-intervention, and post-intervention assessments for the post-assessment model, and all four assessments for the follow-up model) generally supported the ITT results. At follow-up, the SPIN scores differed significantly between the SOPHIE and CAU conditions (*p* < 0.001). In contrast to the ITT analyses, a significant difference between study conditions was also observed at post-assessment (*p* = 0.032). Subgroup analyses by diagnostic condition yielded the same results pattern as in the ITT-analysis (see Supplementary Table 2).

### Qualitative analysis

Out of *N* = 32 eligible adolescents, *n* = 17 participated in the qualitative interview. For this sample, information on demographic and intervention usage is provided in supplementary Table 1. Four main summary domains were identified, detailing adolescents’ experiences with the intervention: (I) therapeutic relationship, (II) factors contributing to or preventing engagement with the intervention, (III) adolescents’ reflection on their symptom and behaviour changes, and (IV) adolescents’ evaluation of the SOPHIE intervention content. Definitions, subdomains and examples are described in Table 7. The relationship with the e-coach varied greatly (i.e., domain I). Some adolescents found it supportive, others perceived it as impersonal like a chatbot, or even as stressful and uncomfortable because they assumed that their progress in the intervention was observed. E-coaches were also seen as providing a clear structure that helped some adolescents to make progress. Additionally, some adolescents appreciated the format of remote support, without the need to visit a therapist.

**Table 7:** Definitions of and examples of all domain summaries and subdomains.

Domain summaries	Definition	Examples
**Therapeutic relationship**	This main theme encompasses the experienced or non-experienced qualities of the relationship with the e-coach (the person conducting the guidance in the SOPHIE programme).	
Burdensome	This subtheme contains experiences of stress and burden. By looking at completed exercises and commenting on them or giving feedback, the e-coach made adolescents feel guilty or pressurised, they were afraid of disappointing the e-coach if they do nothing/something wrong. Furthermore, adolescents explain that they did not know how to interact (are they expected to write back/not?).	Emma (17. 02): “I found it a bit uncomfortable, (…) I hadn’t worked on a module for a week because I was really stressed, I still had school and so that week, I didn’t think about it at all, it was a bit stupid, and then I got a message from the e-coach saying (…) I haven’t completed one, I totally had the feeling of pressure, oh god I’ve forgotten something, I hope they still like me.” Nora (16.02): “Mmh, well, I didn’t really answer because it always made me very nervous to know that someone was looking at what I was working on.”
Supportive	In this subtheme, e-coaches are described as warm, supportive and understanding. Adolescents felt seen and recognised that they are being addressed personally (emotional component). E-coaches give feedback, answer questions, explain how to proceed and are perceived as helpful (structuring component). This structuring goes beyond reminders.	Emily (16.05): “I thought it was good because you could write the e-coach all the time. They replied pretty quickly and gave me tips. I thought that was good. So, I also thought it was good that everyone was supported during the time and that they didn’t just say ‘yes, go through the 6 modules and if you have any questions well that’s too bad, but we can’t help you’, but that you were supported, that you could always turn to the e-coach, yeah.” Lea (17.07): “Ehm, so I found that helpful. (Alessja: Mhm) And I found it nice that a person tells you that you can continue [with the next module] and not just an automatic message. I found that a more personal thing.”
Unpersonal	This subtheme captures the experience of having “no” relationship. Adolescents perceived the interaction as impersonal and wonder whether it is a Chatbot/artificial intelligence. They perceive no contact person (as compared to face to face, for example).	Emma (17.02): “Well, I didn’t really see a relationship if I’m honest, I didn’t know exactly, I didn’t want to answer because I didn’t want to cause anyone any stress, so yes, I thought it was a bit, they always wrote me the same thing and then I thought it was a bit like a bot, like a not a person or something.”
**Factors contributing to or preventing engagement with the intervention**	This main theme contains factors that contribute to or prevent adolescents from engaging with the intervention. In contrast to adherence that assesses engagement with a predefined (amount of content), this theme encompasses subjective factors that support adolescents’ engagement with the intervention	
Problem awareness and motivation to change (initial Engagement)	In this subtheme, adolescents recognize their problems, symptoms or challenges and would like to learn how to handle these situations differently with support of the intervention.	Anja (16.06): “Ehm I just realised that I have social anxiety and that it would be cool to try to do something about it. And then I realised that there was this study and the things on the videos applied to me and I thought it was a good opportunity to start something.” Emma (17.02): “Ehm, so mainly my fears, firstly that I can’t participate verbally and then that I might be afraid to show myself to others, that I’m afraid of what others might think about me and to find out what’s going on in my body and why I’m afraid, what’s causing it.”
Interest in the content/topic supports engagement	This subtheme entails adolescents interested in the programme, the topics or research as a reason to engage with the intervention (not necessarily linked to own situation).	Anja (16.06): “Ehm I just wanted to try it out and see if it helps me [and] I was interested - I don’t know.” Sophie (15.05): “Ehm honestly, I was just curious about what was still to come. As for the ending, the way it ends, so to speak.”
Experienced self-efficacy leads to continuing engagement	This subtheme contains recollections of symptom improvements due to the SOPHIE programme. Adolescents experience themselves as self-effective with the support of the programme and motivated to experienced symptom change and indicate this as the reason to keep using it.	Nora (16.03): “Mmh, I usually worked on one [module] every week, so I usually had time to realise that the modules really help, and I always found it very interesting to learn about anxiety and that motivated me to keep going.” Lea (17.07): “I would also say that I saw a small improvement, that also motivated me to keep going.”
Design, structure and reminders support engagement	This subtheme entails references to the module-based structure (one per week), the reminders by the e-coaches or parents support that supported adolescents’ engagement with the intervention	Emily (16.05): “[I liked] that you could actually click through the programme every week and you could write down things that you noticed or that happened every week and then you could see them again and then see whether you got better at them or whether they stayed the same.” Lara (11.10): “Mostly my mum also told me that I still had to do it or sometimes I was told by my e-coach so I received a message and it said that I still forgot to do one module or something.”
Online delivery promotes autonomy and flexibility	In this subtheme, adolescents appreciate the anonymity and independence an online intervention holds for them. Some adolescents mention the fact that they do not have to meet a person or travel anywhere, others appreciate that the programme is at no cost, the freedom to decide for themselves which exercises they want to do, the time flexibility and the sustainability (the intervention can continue to be used after official completion).	Elin (15.03): “I went to a therapist, but it didn’t work properly because I didn’t feel comfortable with the person. And then I thought I’d try it myself first. And that it was still somewhat guided, I thought that was a very good option.” Sara (16.11): “I found it very interesting that it’s an online programme and you don’t have to go somewhere and see a therapist or anything like that. And yeah, I thought it was good that different exercises were offered, and you didn’t always have to try them all.”
Other commitments and lack of time prevents engagement	In this subtheme, adolescents describe that they did not use or forgot to use the online programme due to exam stress, too little or no time, or other leisure activities such as holidays.	Anna (16.10): “I just, I think I had almost no time two weeks before the end because there was so much going on. At school and everything. And I don’t know, maybe I could have divided it up differently or something, I had the feeling that I should do something, but at the same time I didn’t find any time.” Elin (15.03): “I’m a very forgetful person, which means I forget a lot. Even things that I actually know I still have to do.”
**Adolescents reflect on their symptom and behaviour changes**	In this main theme, adolescents reflect on possible subjective changes in their behaviour and symptoms.	
Adolescent recount observable implementations in their daily life	In this subtheme, adolescent recount observable implementations in their daily life. They describe how they apply content (e.g. strategies, exposure exercises) in their everyday life; thereby, this theme is focused on implementation and not on understanding psychoeducational content.	Emma (17.02): “I was able to come up with my own phrases [words of encouragement] and I also got input on what I could use for phrases. I was able to recall [them] in my head the moment the panic raised. I thought that was really cool that I was sitting there in class and when the panic started, I sayed to myself ‘I’m enough and I can do it! And if not, there are other opportunities, no stress, it’s all good’.” Mia (15.07): “Ehm. So, it certainly worked well for me to plan situations like that [exposure exercises] and then put them into practice. It also helped to know, okay, now I’m going to do this and setting a date also helped.”
Difficulties implementing changes in daily life	This subtheme contains accounts of adolescents of difficulties in implementing what they had learnt in the intervention into their everyday lives. Some adolescents also indicate that they would need additional support.	Anja (16.06): “The SOPHIE-programme definitely gave me some tips. I don’t know if I’m good at applying them because it’s sometimes difficult to think clearly in this situation. But I definitely got some tips.” Linn (15.05): “I thought the fear pyramid was quite good, because you could show yourself what you are actually most afraid of and what is the worst thing for you. Ehm, but it was just a bit too difficult to put into practice, especially alone, I’d say. Because you are not able to ask questions.”
Subjective Symptom Change	This subtheme captures adolescents’ reflections on their subjective changes in symptoms	
Personal problem(s) worsened or remain unchanged	This subtheme contains accounts of adolescents who report a lack of improvement or worsening of their symptoms or problems.	Louise (16.10): “Certainly on the one hand a few messages that I took away from the modules, e.g., thinking of a [safe and happy] place, but I think the whole programme, hadn’t helped me that much personally. I think it helped that I became clearer about what I could do better and that I now know what to do next and how to work on it. So I think it has helped me indirectly, maybe that I now have this motivation to change something and yes, even if the exercises didn’t work for me personally, I do think that it has triggered something in me that I want to improve.”
Personal problems ameliorated	In this subtheme, adolescents describe subjective improvement in symptoms or report on fewer problems.	Mia (15.07): “Ehm, I have two main problems, so to speak. One is giving presentations and standing in front of the class and speaking up and the other is talking to new people or generally starting conversations with strangers. And yeah, I think starting conversations and getting to know strangers has got better now and with the presentations, I haven’t had the chance to try it out yet because we haven’t had any presentations at school yet. But I think it will certainly get better.”
Adolescents think they need more time to experience significant change	In this subtheme, adolescents explain that they need more time before an improvement could take place.	Anja (16.06): “I have the feeling that it did get better. But I also have the feeling that I need more time so that I can apply the things from the programme even more (…) and have the confidence to do the things.”
**Adolescents’ evaluation of SOPHIE intervention content**	This main theme captures adolescents’ reflections on the SOPHIE intervention content.	
Psychoeducational content was received as instructive, informative, understandable.	In this subtheme, adolescents report that videos, audios and texts were understandable, and that it was helpful to them to learn more about the topic. They report that content helped them understand their challenges and situation better.	Emily (16.05): “I thought it was good that there were videos in between so you could watch them again, then you understood what you had read. I thought it was very good that I could recognise myself a bit in the videos. That they were adolescents and not adults.” Lea (17.07): “Ehm, so certainly a lot of people feel like me. And that if you keep at it, there are possibilities to change.” Lucie (16.05): Ehm so in any case I got more information and examples from others of what [social anxiety] can look like and how you can observe it in yourself and that’s definitely made me a bit more confident. And I know better how to deal with it.”
Psychoeducational content was not relevant, too theoretical or not understandable for adolescent.	This subtheme contains descriptions of adolescents that the psychoeducational intervention content was not relevant to them and their situation. A few adolescents also report that they had difficulties to understand it.	Noah (16.04): “Mmh, I just had difficulties that I didn’t need it [the information] for myself because, as I said, I made progress relatively quickly at the beginning and developed further in that sense and yeah then you just didn’t do it [the programme] somehow.” Interviewer to Neele: “If you didn’t understand something, could you ask your parents?” Neele (11.00): “Exactly, my parents were usually able to help me, or they googled it. And if that didn’t work, then I went to my e-coach. But mostly my parents knew the solution.”
Exercises and practical applications were experienced as helpful and suitable	This subtheme captures adolescents’ descriptions of how they found the exercises and strategies useful, helpful and applicable for themselves.	Louise (16.10): “I thought that was good, so if I really made an effort and wrote in the logbook, then it stuck [in my mind]. And that’s why I think it’s good. I also thought it was good to look back and remember things. I think if nothing had happened after the exercise, I would have forgotten it even quicker than I have now.” Interviewer: “So, the anxiety pyramid was the thing that helped you the most, why would you say it was so helpful?” Neele (11.00): “Yes, I was able to make a timetable and write down very specifically that I would do it this way and that way. And that’s what it said on the timetable, then I must do it like this.”
Exercises and practical applications were experienced as not suitable for adolescent’s specific life situation.	In this subtheme, adolescents report that they cannot use the proposed exercises in their everyday life. In contrast to implementation difficulties, they don’t indicate to need additional support, but rather that the exercise does not fit their everyday life (e.g. the duration of the relaxation exercise is too long).	Mia (15.07): “So these exercises took quite a long time and you have to be able to apply them very briefly in these [social] situations, there wasn’t enough time for me to be able to do them. Ehm exactly, that was certainly the difficulty.” Emma (17.02) on relaxation exercises “Yes, exactly. It was always about 10 minutes long and I really haven’t had that much time recently, especially with exams and school, so I’ve never really found time to lie down or sit down for 10 minutes and listen to it. I think I’m also a very impatient person and then I didn’t really manage to pick myself up and say sit down now, I pushed it to the back of my mind and only did it once or twice, which was a bit of a shame.”

Adolescents discussed further factors contributing to their engagement with the intervention (i.e., domain II). Awareness of the problem and motivation to change played key roles, especially in the early phase of the intervention. Interest in the content and personal improvements acted as motivators to continue. Structure of the program, including reminders by e-coaches or parents, or module structure, were perceived as helpful to plan and organise the use of the intervention over the eight weeks. The most common barrier to engagement was lack of time or forgetting.

When adolescents reflected on changes in their symptoms and behaviour (i.e., domain III), their responses diverged: some reported symptomatic improvement, others noted partial improvement but felt that more time was needed, and some reported no improvement or worsening of their problems. Some noticed tangible improvements in daily life, while others struggled, especially with behavioural tasks such as exposure exercises. Although they could understand the importance of doing exposure exercises, many found it challenging to implement them independently and some indicated that they would need additional support.

The content of the intervention (i.e., domain IV) was generally understandable and relevant. Some felt seen and understood because of examples and videos that were targeted to them (i.e., problems, age group). Younger adolescents sometimes struggled to understand the psychoeducational content but could seek help from their parents. Exercises were generally regarded as helpful and suitable although some found it difficult to adapt them to their own lives and thus did not engage in them.

## Discussion

The primary aim of the RCT was to evaluate the efficacy of the online intervention SOPHIE in reducing social anxiety in adolescents. While no significant difference between the intervention and control group was found post-intervention in social anxiety, a significant difference emerged at 3-month follow-up with a medium to large effect size. Subgroup analysis showed significant differences with a large effect size for the subclinical group (i.e., indicated prevention) at follow-up but not for the SAD group (i.e., treatment). Secondary outcomes, such as depression, general anxiety, social fear and avoidance, and quality of life, showed significant improvement post-intervention with small to large effect sizes. However, these improvements were not sustained to follow-up. The intervention group significantly improved in level of social (but not role) functioning at post- and follow-up assessments with small to medium effect sizes. Guardians reported no significant changes in their child’s social anxiety.

Adolescents in the intervention group were interviewed to gain information on their experiences using the SOPHIE programme. In the qualitative analysis four main topics were explored (1) therapeutic relationship, (2) factors that contributed to and prevented engagement with the intervention, (3) reflections on symptom and behaviour change, and (4) adolescents’ evaluation of the SOPHIE intervention content.

The non-significant intervention effect on social anxiety after the intervention is inconsistent with previous online intervention studies in adolescents with SAD^34–36^. However, social anxiety was significantly reduced at follow-up suggesting a delayed response. One possible explanation is that adolescents may require more time than an eight-week intervention period to practice the learned strategies and to implement the acquired knowledge and skills to everyday life^25,43^. This aligns with previous evidence that cognitive and behavioural gains from anxiety treatments in adolescents continue to develop after the intervention period and can show a delayed response^26,44^. A further explanation for the delayed response could be drawn from the Cognitive Model of Social Phobia^30^ which posits that social anxiety is maintained by mechanisms such as avoidance behaviour^29^. In this study, avoidance behaviour significantly decreased at post-intervention but not at follow-up suggesting early reductions may have led to later symptom reduction. This temporal pattern, in which changes in maintaining mechanisms precede social anxiety symptom changes, has been observed in another study^,^^45^. Thus, targeting the proposed mechanism in interventions might be needed to facilitate subsequent improvements in social anxiety.

Notably, the significant difference in social anxiety at follow-up was mainly driven by the reductions in the subclinical but not in the clinical group. This is in line with previous research on indicated prevention of social anxiety symptoms in face-to-face settings, specifically in group formats^46^. In contrast, indicated prevention delivered online showed significant small effects for depressive but not for anxiety symptoms in meta-analyses^19,47^. This inconsistent evidence may partly be due to varying recruitment strategies in identifying adolescents at risk for the development of a mental disorder and that social anxiety symptoms specifically are poorly recognised by affected adolescents and their social context^2,48,49^ resulting in heterogenous samples with different needs, problem awareness and treatment motivation^50^.

The clinical SAD-group showed only little improvement in this study. This may be due to high rates of comorbidities and impaired functioning that characterized our clinical sample. Consequently, some participants may have been too burdened to benefit from a guided self-help approach with a focus on social anxiety. This may also explain the rather low SAD-remission rates found in our study and a similar online intervention for adolescents^34^. These participants may require more intensive treatments addressing multiple problems. Additionally, some adolescents participated simultaneously in psychotherapy in a face-to-face setting, suggesting a need for a coordination of these interventions using blended formats^51^.

Adolescents were primarily enrolled through their guardians, highlighting their essential role in adolescents’ help-seeking behaviour^52^. However, guardians reported no significant changes in social anxiety at all assessment points, unlike adolescents. This may be explained by discrepancies generally found between adolescent and guardian reports, particularly pronounced in internalised disorders^53–55^.

The SOPHIE-intervention led to positive effects on social functioning. This effect is in line with previous RCTs of online interventions for SAD in adolescents that also reported significant improvements in global functioning post-intervention^29,34,35^. In this study, social but not role functioning increased significantly at both post-intervention and follow-up, suggesting a generalisation effect on adolescents’ social environment. This highlights that the intervention may have differential effects on everyday life and may be especially relevant for the social behaviour of adolescents. Unchanged role functioning may indicate that improvements in academic and vocational functioning takes more time to manifest, emphasizing the importance of early interventions to prevent negative long-term impacts^56^.

Although significant differences in depression, general anxiety and quality of life were found at post-assessment, these effects have attenuated until follow-up. Additional interventions targeting specific disorders or a transdiagnostic approach may be warranted to improve these effects over the long term, particularly in the presence of comorbidities^57^. These results are in line with other studies on online interventions for adolescents that demonstrated no significant effects of anxiety-focused interventions on depression and quality of life^58^.

Adolescents experienced the SOPHIE-intervention as beneficial and supportive but challenging like previous qualitative results in context of online interventions for adolescents with SAD^39^. Exposure exercises were perceived as the most difficult ones. Thus, guidance could be specifically intensified during the planning and implementation of these exercises. This would support adolescents at the right time without generally restricting their autonomy. This seems especially relevant because some adolescents felt controlled and pressured by the regular weekly guidance, suggesting the need for varied guidance formats. The effect of letting adolescents choose their preferred guidance format at the start of the intervention could be investigated in future studies ^e.g.,^^59^.

Most adolescents noticed initial improvements in symptoms during the intervention but needed more time for implementing it to their everyday lives, which is consistent with efficacy results on social anxiety that were only significant at follow-up. If content was not directly relevant to their situation, they reported implementing it less often or not at all. Additional guidance on demand could be helpful to support to better tailor the exercises and modules to the very specific needs of the participants ^i.e.,^^39^. This may improve the implementation of online interventions and their efficacy. To this aim, future research could make use of techniques such as the think-aloud method to explore adolescents’ real-time use of the intervention to gain more detailed user-led information on their specific needs^60,61^.

These results should be interpreted considering some limitations. The sample was a self-selected group of adolescents who had expressed an interest in the SOPHIE intervention which may limit the generalisability of the results. In addition, the targeted sample size for the subclinical group was not reached. Nonetheless, post hoc power analyses indicated that the study still had sufficient power to detect effects in the overall, clinical and subclinical group at follow-up. Despite this, the limited sample size may have constrained the ability to detect smaller effects that could emerge in a larger sample with more participants exhibiting subclinical symptoms. Importantly, the use of a clinical interview to exclude participants with a past and present diagnosis of a SAD to define the subclinical group represents a methodological strength, and future research should aim to better recognise adolescents with subclinical levels of social anxiety and investigate larger-scale SAD-specific prevention programs. As the intervention effects only became evident at the time of the 5-month follow-up, no conclusions can be made about longer-term improvements and the full impact of the intervention; to evaluate this a longer follow-up period in future studies is needed. Adolescents in the qualitative study were mostly committed to the intervention, thus limiting the in-depth exploration of barriers to intervention engagement. Future studies could capitalise on the strengths of mixed-method approach and look even more in-depth at the experiences of adolescents, especially those who make less use of the intervention. Moreover, the investigation of various means of support for adolescents before and during the usage of the intervention (e.g., by including modules tailored to their needs, personalised formats of guidance)^36^ may further increase the efficacy of intervention. Additionally, introducing exposure exercises early during the intervention could be beneficial, particularly considering decreasing adherence over time^46^.

## Conclusion

This study adds to previous evidence on the efficacy of disorder-specific online interventions for social anxiety based on conceptual models such as the cognitive model^30^ and supports the longer-term efficacy of online interventions for social anxiety, particularly for subclinical social anxiety. It further suggests new avenues to personalise future interventions. Leveraging online interventions has the potential to substantially improve mental health in adolescence and beyond.

## Method

### Study design

This RCT investigated the effects of the online intervention SOPHIE compared to a care-as-usual (CAU) control group in adolescents with subclinical social anxiety or SAD. The trial had a 2 × 2 × 4 design: experimental condition (SOPHIE vs. CAU), diagnostic condition (SAD vs. subclinical social anxiety), and repeated assessments (baseline, mid-intervention at four weeks, post-intervention at eight weeks, and follow-up at five months after randomisation). Qualitative interviews were conducted post-intervention with participants in the intervention condition.

The trial was registered in clinicaltrials.gov (date of first registration: 04/03/2021; registration number: NCT04782102) and was conducted following the declaration of Helsinki. It was approved by the Ethics Committee of the Canton Bern in March 2020 and the amendment to implement the qualitative study in May 2022 (CEC Bern, Project ID 2020–02501). Detailed information (except for the qualitative interview) is provided in the study protocol^41^. The trial further included assessment on potential mediators and moderators of the effects of the intervention which will be published separately.

### Participants

#### Sampling strategy

A self-selected sample of participants was recruited in the German speaking countries (Switzerland, Germany, Austria, Liechtenstein) from August 2021 to August 2023. To reduce self-selection bias and support the sampling of adolescents with subclinical social anxiety, a recruitment strategy was established to distribute information about social anxiety, SAD and the possibilities of digitally offered help among three target groups: adolescents, their caregivers and their professional network. All information and promotional materials were adapted to the target groups’ needs and brought to them by different means of communication. Adolescents were targeted by advertisements on social media channels (i.e., Instagram and TikTok), school visits, workshops, flyers and posters in buildings frequented regularly by adolescents (e.g., youth centres). Caregivers were targeted by information postings in educational magazines, newsletters from parents’ associations, internet forums and advertisements on Google. The professional network adolescents are embedded in (i.e., teachers, social workers, school mental health specialists, therapists and primary physicians) was informed by collaborations with partner organisations, workshops and information talks given by the first and last authors.

Participants for the qualitative interviews were recruited from the intervention group of the RCT beginning in July 2022, following receiving ethical approval for the qualitative component of the study. After this time point, 36 adolescents were randomised to the intervention group. Of these, 4 withdrew consent prior to the post-intervention assessment, leaving 32 eligible participants. All 32 were invited to participate in the qualitative interviews, and 17 participated. See the supplementary material for an analysis of differences between adolescents who participated in the interview and those who did not.

#### Eligibility criteria

Inclusion criteria required participants to be 11 to 17 years old, understand German, have access to a device connected to the Internet, and score 16 or higher on the Social Phobia Inventory (SPIN). This threshold was chosen following previous research^62^. Exclusion criteria were a known diagnosis of autism spectrum disorder, acute suicidality at baseline, and a past diagnosis of SAD for the subclinical group. All participants who consented and were randomised to the intervention group were eligible for the qualitative interview.

### Procedure

Informed consent (14 to 17 years) and informed assent with guardian consent (11 to 13 years) were obtained. Baseline assessment included questionnaires and a diagnostic telephone interview. Participants were then randomly allocated to the intervention or CAU group via stratified block randomisation based on diagnostic status (SAD vs. subclinical social anxiety). Randomisation parameters (number of groups, allocation ratio (1:1), and block size) were set up by the first author using Qualtrics embedded in the last baseline assessment. The study team and participants were informed of their group allocation after randomisation. Participants filled in questionnaires three times (four weeks, eight weeks, five months) after randomisation. Post-, and follow-up assessments also included a diagnostic telephone interview with interviewers blinded to the study condition. Participants were allowed to seek additional mental health support.

#### Online intervention SOPHIE

The eight-week online intervention SOPHIE is based the Cognitive Model of Social Phobia^30^ adapted to specific needs of adolescents^29^ and existing evidence-based online interventions for adults with SAD^63–69^. It consists of 8 modules^41^: introduction and goal setting, six modules dedicated to the maintaining mechanisms of the Cognitive Model^30^including negative automatic thought processing, self-focused attention, and avoidance and safety behaviour, and a final module with a short repetition and outlook. Adolescents were guided by trained and supervised graduate students in Psychology^41^. For more information of the development of the intervention and a detailed description of the modules, please consider the study protocol^41^.

#### Care-as-usual (CAU) control condition

Participants in the CAU group were only contacted regarding assessments and received access to SOPHIE after the follow-up assessment. They received no specific intervention and were free to access all kinds of support services. The use of other support services was assessed at post-assessment.

### Materials

#### Primary outcome

The self-report scale *Social Phobia Inventory* (SPIN; original English version^70^; German version^71^) is an internationally used and recommended^70^ continuous measure assessing 3 dimensions of social anxiety (i.e., fear, avoidance, and physical symptoms). In each of the 17 items, symptoms of the past week are rated on a 5-point Likert scale (scored from not at all (0) to extremely (4); total range: 0–68)^72^. An example item is “I avoid talking to people I don’t know”. In adolescent samples, the SPIN has shown good psychometric properties to detect subthreshold and threshold SAD^73,74^ and demonstrated good test-retest reliability (*r* = 0.81) and internal consistency (Cronbach’s *α* = 0.89)^74^ and evidenced good convergent and discriminant validity^75^. The SPIN value at post-assessment was defined as the primary outcome.

#### Secondary outcomes

##### Self-report measures

At baseline, mid-intervention, post-intervention and follow-up, general anxiety symptoms were assessed with the *Generalized Anxiety Disorder-7* (GAD-7)^76,77^ depressive symptoms with the *Patient Health Questionnaire-9 for Adolescents* (PHQ-A)^78^ fear and avoidance with the *Social Anxiety Scale for Adolescents* (SAS-A)^79,80^ quality of life with the *KIDSCREEN-10*^81^, and self-esteem with the *Rosenberg Self-esteem Scale* (RSES)^82^.

The GAD-7 questionnaire (original English version^83^; German adolescent version^76^) measures self-reported frequency of anxiety symptoms with 7 items rated on a 4-point Likert scale scored from not at all (0) to nearly every day (3) (total range 0–21). An example item is “Feeling nervous, anxious or on edge”. In adolescents, the questionnaire has demonstrated good psychometric properties with good internal consistency (Cronbach’s *α =* 0.91) and construct validity^77^.

The PHQ-A (original adolescent English version^78,84^; German version^85^) assesses frequency of depressive symptoms over the last 2 weeks in adolescents with 9 items rated on a 4-point Likert scale from not at all (0) to nearly every day (3) (total range 0–27). A sample item is “Little interest or pleasure in doing things”. This self-report questionnaire shows good psychometric properties for detecting depression in adolescents^78,84^ and a pooled Cronbach’s *α =* 0.86 across the life-span^86^.

The SAS-A (original English version^79^; German version^87,88^) measures fear of negative evaluation and social avoidance in adolescents with 18 items answered on a 5-point Likert scale from not at all (1) to all the time (5) (total range 18–90). A sample item is “I worry about what others say about me“. The measure has shown good validity and reliability in both clinical and nonclinical samples and the internal consistency for the total scale was Cronbach’s *α =* 0.91^79,80^.

The KIDSCREEN assessments were simultaneously developed in different European countries and languages including German^89^. The KIDSCREEN-10 assesses health-related quality of life with 10 items answered on a 5-point Likert scale rated from never/not at all (1) to always/extremely (5) (total range 1–50). A sample item is “Have you felt fit and well?”. The scale has demonstrated good psychometric properties and reported a Cronbach’s *α =* 0.82^81^.

The RSES (original English version^82^; German version^90^) assesses self-esteem in 10 items on a Guttman scale rated from strongly disagree (0) to strongly agree (3) (total range 0–30). A sample item is: “On the whole, I am satisfied with myself”. In this study the adolescent version of the questionnaire was used. This version has shown good psychometric properties with a Cronbach’s *α =* 0.81^91^.

##### Clinician-rated interviews

Adolescents were assessed for past and current mental disorders at baseline according to the Diagnostic and Statistical Manual of Mental Disorders-5 with the *Diagnostic Interview for Mental Disorders for Children and Adolescents*(German: Kinder DIPS;)^92,93^. At post-intervention and follow-up, only the section for SAD and diagnoses met at baseline were assessed. Global functioning was assessed at baseline, post-intervention and follow-up with the *Global Functioning Social and Role Scale*, a structured interview yielding a score from 1 to 10 for each subscale^94,95^.

##### Adherence

Adherence was operationalised through the extent to which the online intervention was used. The number of finished modules, of completed exercises, and the time spent in the online intervention were recorded automatically.

##### Guardian report

If guardians provided their e-mail address, they rated their child’s social anxiety with the *Parents’ Questionnaire on Social Anxieties in Childhood and Adolescence* (German: Elternfragebogen zu sozialen Ängsten im Kindes- und Jugendalter (ESAK); range 0–54)^96^ at baseline, mid-treatment, post-intervention and follow-up.

#### Assessment of negative effects and satisfaction with intervention

Negative effects of the intervention were assessed post-intervention with the *Inventory for recording negative effects in psychotherapy* (INEP)^97,98^ and satisfaction with the intervention with the *Client Satisfaction Questionnaire* (CSQ-8; mean value between 1–4)^99^.

#### Demographic information

Adolescents reported their age, gender, nationality, native language and living situation during the baseline telephone interview. Socioeconomic status was measured with the *Social Affluence Scale* (range 0–12)^100,101^. Guardians reported their level of education, employment status and living situation during their first assessment.

#### Qualitative interview

The qualitative assessment consisted of a semi-structured interview. The questions covered topics that were based on the stages adolescents move through when participating in an intervention study: awareness of their problems and of digital help, consideration of and expectations towards study participation, and experiences during the intervention^102,103^. Regarding the intervention, helpfulness of individual modules and experiences with guidance were explored. Questions were open-ended, but the interviewer could provide a structured scale (i.e., from very helpful to not at all helpful) as a starting point for a more detailed discussion. The final interview guide can be found in the supplementary material.

### Analysis

#### Sample size

The initial sample size was determined in two a-priori analyses (SAD-group and subclinical group) for the primary research question in G*Power^104^ based on an intention-to-treat (ITT) approach and repeated measures analyses of variance (ANOVA). The analysis for the SAD group was based on small-to-moderate effect sizes (Cohen’s d =.35)^105,106^ and the analysis for the subclinical anxiety group on a small effect size (Cohen’s d =.20)^47,107–109^. We assumed an α-level of 5%, a power of 80%, and a correlation between measurements of *r* = 0.4. The targeted sample size was *N* = 222 including *n* = 56 adolescents with SAD and *n* = 166 adolescents with subclinical social anxiety.

As the targeted sample size for the entire sample and the subclinical anxiety group was not reached, post hoc power analyses were conducted using G*Power. The parameters matched those of the a priori analysis, with an alpha level of 5% and an assumed correlation between repeated measures of *r* = 0.40. For the main analysis (effect size d = 0.67, *N* = 133), the calculated power was 0.97. Similarly, the power was 0.99 for the SAD group (d = 1.21, *n* = 93) and 0.99 for the subclinical anxiety group (d = 1.53, *n* = 40).

#### Efficacy analysis

All participants were analysed using the ITT approach. Baseline differences were assessed with Chi-square tests for nominal data and independent t-tests for continuous data. Patterns of missingness in primary and secondary outcomes were visually inspected. The possible influence of demographic variables, study condition, and baseline assessments on missingness was analysed with Chi-square tests for binary predictors and logistic regressions for continuous predictors.

The primary outcome, difference of social anxiety symptoms between study groups at post-assessment, was analysed using linear mixed-effects models with restricted maximum likelihood estimation with the *lmer* function of the lme4 package^110^ in R (version 2023.12.1 + 402). Linear mixed-effects models were selected because they account for irregular assessment timepoints and dependencies in longitudinal data and provide unbiased estimates under the missing at random (MAR) assumption, using maximum likelihood estimation^111–113^. In the final model, time (baseline, mid-intervention, post-intervention), intervention condition (SOPHIE, CAU), and interactions were specified as fixed effects. Baseline SPIN value was added as a fixed covariate, and participant was included as a random effect to allow for between-person variation. The same model was used to analyse intervention effects on secondary outcomes (i.e., general anxiety symptoms, depressive symptoms, quality of life, self-esteem, and guardians’ rating of social anxiety). Follow-up effects were analysed using the same model with all four assessment timepoints included. The SPIN model for post- and follow-up effects was first computed with all participants and then separately in subgroup analyses with participants of the clinical group and the subclinical group. Remission of SAD diagnosis was only assessed in the clinical group, and Chi-square tests were used to compare intervention and control groups at post- and follow-up assessments.

Additionally, sensitivity analyses were performed for the primary outcome SPIN using a per-protocol sample. For the post-assessment model, the per-protocol sample (*n* = 77) included participants who completed all three assessment timepoints: baseline, mid-intervention, and post-intervention. For the follow-up model, participants (*n* = 57) had to complete all four timepoints: baseline, mid-intervention, post-intervention, and follow-up. We used the same models as described above for these analyses.

#### Qualitative data collection and analysis

The qualitative interview was conducted after the intervention with adolescents from the SOPHIE study group via telephone. Individual interviews were audio-recorded and afterwards transcribed verbatim. A reflexive thematic analysis (RTA) approach was chosen due to its flexibility to explore the full range of experiences and to account for subjectively perceived realities^114,115^. For this study, a contextualist standpoint was adopted, allowing us to interpret adolescents’ experiences within their social contexts. A reflexive, iterative analysis process was followed, where themes were co-constructed based on participant experiences, aiming for a nuanced understanding of central ideas and concepts^114,116^. Following the six steps of RTA, authors NW and DH familiarised themselves with the data and coded text parts that reflected experiences of participants during and with the intervention. Both authors discussed and reviewed initial codes to develop first possible themes. Subsequently, the full transcripts were re-coded with the identified themes and subthemes, leading to further adjustments to the themes. A thematic map was created and discussed with the research team. During the theme review process, we found that the interview data was not sufficiently rich to develop themes united by a central concept or idea that were not solely reflecting the topics of the interview guide. Thus, instead of overinterpreting or presenting underdeveloped themes^115,117,118^we focused on creating themes with a shared topic and common points expressed by participants in relation to this topic. This approach led to sub-domains of similar experiences, which we consolidated into domain summaries exploring different facets of each topic. Finally, both authors coded the transcripts again. Domains and sub-domains were exemplified with quotes from the transcripts using pseudonyms for quotation presentation. All quotes were translated from (Swiss) German and minimally altered to enhance clarity while preserving meaning.

##### Researcher reflexivity

Authors NW and DH conducted the analysis. NW led this RCT as part of her doctoral dissertation thereby providing extensive familiarity with the study and the SOPHIE programme. DH was a Psychology master student new to the project and exclusively handled qualitative data. Both approached the data inductively. Nonetheless, NW acknowledged that her involvement in the study might have influenced her perception of adolescents’ experiences with the intervention. Furthermore, the interviews were conducted by telephone without video conferencing. This way of data collection might have influenced the data richness; some adolescents provided only very brief responses, and even after probing further questions, they did not provide much more content. For adolescents with social anxiety, discussing personal experiences over the phone might be challenging, and for the interviewer, the lack of nonverbal cues may have influenced the understanding of their statements.

## Electronic supplementary material

Below is the link to the electronic supplementary material.

Supplementary Material 1

## Data Availability

The data that support the findings of this study are available upon reasonable request. Please contact Prof. Dr. Stefanie Schmidt: stefanie.schmidt@unibe.ch.
