# Supplementary material for: Prevention and treatment of social anxiety disorder in adolescents: mixed method randomised controlled trial of the guided online intervention SOPHIE
Source: Sci Rep. 2025 Jul 11;15:25141. doi: 10.1038/s41598-025-10193-w (PMC12254345; doi:10.1038/s41598-025-10193-w)
Supplement: Supplementary file 1 — Supplementary Material 1 [file 41598_2025_10193_MOESM1_ESM.docx]

Running head: Mixed method evaluation of the SOPHIE online intervention

**Supplementary Materials**

**Prevention and treatment of social anxiety disorder in adolescents: Mixed method randomised controlled trial of the guided online intervention SOPHIE.**

Noemi Walder*^1^, Thomas Berger^2^, Dominique Hürzeler^1^, Emily McDougal^3^, Julian Edbrook-Childs^3,4^, Stefanie J. Schmidt^1^

1. Division of Clinical Child and Adolescent Psychology, University of Bern
2. Division of Clinical Psychology and Psychotherapy, University of Bern
3. Anna Freud, London, UK
4. University College London, London, UK

*Corresponding Author: Noemi Walder, Division of Clinical Child and Adolescent Psychology, Institute of Psychology**,** University of Bern, Fabrikstrasse 8, 3012 Bern, Switzerland.

E-mail: noemi.walder@unibe.ch

**Supplementary Material**

*Supplementary Table 1. Demographic data and intervention usage of adolescents in the qualitative analysis.*

| Name (fictional) | Age at baseline | Gender | Modules completed | Time spent in SOPHIE programme (in minutes) |
| --- | --- | --- | --- | --- |
| Mia | 15.07 | f | 4 | 168 |
| Emma | 17.02 | f | 6 | 84 |
| Sophie | 15.05 | f | 8 | 221 |
| Anna | 16.10 | f | 8 | 275 |
| Emily | 16.05 | f | 8 | 316 |
| Sara | 16.11 | f | 8 | 333 |
| Nora | 16.03 | f | 8 | 385 |
| Anja | 16.06 | f | 3 | 32 |
| Elin | 15.03 | f | 8 | 195 |
| Linn | 15.05 | f | 6 | 33 |
| Lucie | 16.05 | f | 3 | 114 |
| Lea | 17.07 | f | 8 | 398 |
| Noah | 16.04 | m | 4 | 114 |
| Lara | 11.10 | f | 4 | 145 |
| Louise | 16.10 | f | 6 | 188 |
| Neele | 11.00 | f | 8 | 707 |
| Alex | 12.04 | m | 8 | 375 |

**Group differences**

Of 32 eligible participants for the qualitative interviews, 17 participated. We compared the two groups (adolescents who participated vs. those who did not) on adherence measures (i.e., modules completed, time spent in the SOPHIE programme), demographic measures (i.e., age, gender), and clinical measures (social anxiety (SPIN score) and global social functioning at baseline).

Tests of normality using the Shapiro-Wilk test indicated that most variables violated the assumption of normal distribution in at least one group, except for social anxiety. Homogeneity of variances, assessed with Levene’s test, was not significantly violated for any variable. Therefore, non-parametric Wilcoxon rank-sum tests were performed for all variables except social anxiety, for which an independent samples t-test was used.

Significant differences between groups were found for adherence-related variables. Specifically, time spent in the programme (*p* < .0001) and the number of modules accessed (*p* < .0001) were all significantly higher among adolescents who participated in the interviews. No significant group differences emerged for demographic variables such as gender (*p* = .55) and age (*p* = .16), nor for clinical baseline measures including social anxiety (*p* = .52) and global social functioning at baseline (*p* = .95).

In summary, adolescents who participated in the qualitative interviews spent significantly more time in the SOPHIE programme and accessed more modules compared to non-participants, while demographic and clinical baseline characteristics did not differ significantly between groups.

*Supplementary Table 2.* Sensitivity analyses: Per protocol linear mixed-effects analyses for the primary outcome SPIN from baseline to post and from baseline to follow-up with between- and within group effect sizes displayed in Cohen’s *d*.

| **Baseline – Post** | | Coefficients from the linear mixed-effects models (SOPHIE vs CAU) | | | Effect sizes Cohen’s d [95% CI] | | |
| --- | --- | --- | --- | --- | --- | --- | --- |
|  |  | *Estimates* (SE) | t [CI] | p | Between-group | Within-group baseline post | |
|  |  |  |  |  |  | SOPHIE | CAU |
| SPIN | Intercept | 8.46 (1.68) | 3.16 [3.18;13.74] | **0.002** |  |  |  |
|  | Mid-intervention | -2.02 (2.13) | -0.95 [-6.21; 2.18] | 0.345 | 0.23 [-0.23; 0.68] |  |  |
|  | Post-intervention | -4.58 (2.13) | -2.15 [-8.78; -0.39] | **0.032** | 0.55 [0.09; 1.01] | 0.76 [0.26; 1.26] | 0.18 [-0.24; 0.59] |
| SPIN SAD | Intercept | 8.81 (3.52) | 1.65 [-1.14; 12.75] | 0.101 |  |  |  |
|  | Mid-intervention | 0.65 (2.35) | 0.28 [-3.99; 5.29] | 0.783 | -0.14 [-0.68; 0.40] |  |  |
|  | Post-intervention | -3.47 (2.35) | -1.48 [-8.11; 1.17] | 0.141 | 0.28 [-0.11; 0.98] | 0.98 [0.37; 1.60] | 0.49 [-0.02; 1.00] |
| SPIN | Intercept | 13.66 (6.17) | 2.21 [1.32; 26.01] | **0.031** |  |  |  |
| (subclinical) | Mid-intervention | -8.37 (4.42) | -1.89 [-17.22; 0.48] | 0.063 | 0.39 [0.73; 0.05] |  |  |
|  | Post-intervention | -7.32 (4.42) | -1.65 [-16.16; 1.53] | 0.103 | 0.31 [-0.04; 0.65] | 0.43 [-0.46; 1.31] | -0.41 [-1.19; 0.37] |
| **Baseline – Follow-up** | |  |  |  |  | Within-group baseline follow-up | |
| SPIN | Intercept | 10.44 (3.32) | 3.15 [3.90;16.98] | **0.002** |  |  |  |
|  | Follow-up | -9.44 (2.51) | -3.76 [-14.39; -4.48] | **<0.001** | 1.06 [0.50; 1.61] | 1.61 [0.97; 2.25] | 0.50 [0.00; 1.00] |
| SPIN SAD | Intercept | 4.27 (4.17) | 1.02 [-3.98; 12.51] | 0.308 |  |  |  |
|  | Follow-up | -5.43 (2.92) | -1.86 [-11.20; 0.35] | 0.065 | 0.70 [0.05; 1.35] | 1.77 [1.00; 2.54] | 1.04 [0.40; 1.69] |
| SPIN | Intercept | 18.83 (6.60) | 2.85 [5.63; 32.02] | **0.006** |  |  |  |
| (subclinical) | Follow-up | -17.35 (4.78) | -3.63 [-26.92;-7.78] | **0.001** | 2.00 [0.86; 3.16] | 1.73 [0.50; 2.96] | -0.30 [-1.14; 0.54] |

*Notes. All linear mixed-effects models included the baseline value of the SPIN as a fixed covariate and a random effect of participants. SPIN = Social Phobia Inventory; SAD = diagnostic group including adolescents with a social anxiety disorder at baseline; subclinical = diagnostic group including adolescents with subclinical levels of social anxiety at baseline.*

*Supplementary Material. English translation of the qualitative interview guide*

**Interview**

1. Greetings

*"Hello XY, I am *interviewer* from the SOPHIE study team. We have an appointment today for a follow-up telephone interview. Thank you for taking the time to do this. As already announced by email, I would like to ask you about your opinion and experience with the SOPHIE online programme and study participation."*

2. Inform about recording

*"To concentrate fully on you during the interview, I would like to record our conversation. After the interview, I will listen to this recording and write everything down to have the interview in writing. All information that could be traced back to you will be removed. Afterwards, the recording will be irrevocably deleted. Do you have any unanswered questions?"*

3. Information about the interview

*"The interview will take about 20 to 30 minutes; this depends on how much you want to tell me. There are no right or wrong answers here. I am interested in your opinion and experience of the online programme and participation in the study. If you don't understand a question, you can always ask. Do you have any questions up to this point?*

*I will start the recording now, is that okay with you?"*

Start recording.

First, I will ask you some general questions about the study, then I will ask you some specific questions about the online programme.

1. How did you become aware of the study and the online programme?
2. Reasons for Participation:
   1. What were three reasons for you to participate in the study?
   2. Could other reasons be the online programme or the lottery? (Only ask if not addressed in 2a))
3. Expectations:
   1. What were your hopes for participation?
   2. Where were they fulfilled and where not?
4. At the beginning, you could give your e-mail address to show you’re interested in participating in the study, whereupon the study information was sent to you, and you could sign the consent form.
   1. How did you feel about this process?
   2. What support would you have liked?
   3. Do you have any suggestions for improvement?
5. If you had questions, did you know where to get in touch?
   1. If no: What would have helped you?
6. How helpful was it for you when we reminded you of questionnaires? *if participants has trouble finding an answer, give the following possibilities* From 1 to 3; 1 would not be helpful at all, 2 would be helpful and 3 would be very helpful.
   1. Why?
7. How did you find the short surveys via the SEMA3 app?
   1. What were the difficulties (in not completing them)?
   2. How did you deal with these difficulties?
   3. What would you have liked / could have been improved?
8. Next, I would like to ask you some questions about the online programme.
   1. What do you take away from the Sophie online programme?
   2. What did you benefit from the most?
9. Which of the topics helped you the most? If you put them in order, which topic block was the most helpful, which was the least helpful?
   1. Automatic thoughts
   2. Self-image
   3. Self-focused attention
   4. Reality tests (i.e., exposure exercises)
   5. Self-confident behaviour (i.e., social skills)
   6. Why did you choose this order?
10. Programme Design:
    1. How would you rate the amount of text? From 1-3; 1 would be too little, 2 just right and 3 too much text.
    2. How would you rate the amount of videos? From 1-3; 1 would be too few, 2 just right and 3 too many videos.
    3. How would you rate the amount of exercises? From 1-3; 1 would be too few, 2 just right and 3 too many exercises.
11. How helpful did you find...?
    1. The relaxation exercises?
    2. The logbook?
    3. The reality tests?
12. Are there things you did not like about the online programme?
    1. Why? What suggestions would you have for improvement/change?
13. How many of the 8 modules have you completed in total?
    1. If you did not complete all of them, what were the reasons?
    2. What motivated you to keep going?
14. How did you feel about the relationship with the e-coach?
    1. What behaviour from the e-coach was helpful?
    2. What would be helpful?

**CONSORT-SPI 2018 Checklist**

| **SECTION** | **ITEM #** | **CONSORT 2010** | **CONSORT-SPI**  **2018** | **REPORTED ON PAGE #** |
| --- | --- | --- | --- | --- |
| **TITLE AND ABSTRACT** | | | | |
|  | 1a | Identification as a randomised trial in the title^§^ |  | p. 1 |
|  | 1b | Structured summary of trial design, methods, results, and conclusions (for specific guidance see CONSORT for Abstracts)^§^ | Refer to CONSORT extension for social and psychological intervention trial abstracts | p. 2 |
| **INTRODUCTION** | | | | |
| Background and  Objectives | 2a | Scientific background and explanation of rationale ^§^ |  | P 4-6 |
|  | 2b | Specific objectives or hypotheses ^§^ | If pre-specified, how the intervention was hypothesised to work | p. 6 |
| **METHODS** | | | | |
| Trial Design | 3a | Describe of trial design (such as parallel, factorial), including allocation ratio ^§^ | If the unit of random assignment is not the individual, please refer to CONSORT for Cluster Randomized Trials | p. 6/7 |
|  | 3b | Important changes to methods after trial commencement (such as eligibility criteria), with reasons |  | Sample size for subclinical group was not reached; p. 15 |
| Participants | 4a | Eligibility criteria for participants^§^ | When applicable, eligibility criteria for settings and those delivering the interventions | p. 8 |
|  | 4b | Settings and locations where the data were collected |  | p. 7-8 |
| Interventions | 5 | The interventions for each group with sufficient details to allow replication, including how and when they are actually administered ^§^ |  | p. 9 |
|  | 5a |  | Extent to which interventions were actually delivered by providers and taken up by participants as planned | p. 17/18 |
|  | 5b |  | Where other informational materials about delivering the intervention can be accessed | p. 9 |
|  | 5c |  | When applicable, how intervention providers were assigned to each group | N/A |
| Outcomes | 6a | Completely defined pre-specified outcomes, including how and when they were assessed^§^ |  | p. 9 |
|  | 6b | Any changes to trial outcomes after the trial commenced, with reasons |  | N/A |
| Sample Size | 7a | How sample size was determined^§^ |  | p. 12 |
|  | 7b | When applicable, explanation of any interim analyses and stopping guidelines |  | N/A |
| **RANDOMISATION** | | | | |
| Sequence  generation | 8a | Method used to generate the random allocation sequence |  | p. 8 |
|  | 8b | Type of randomisation; detail of any restriction (such as blocking and block size)^§^ |  | p. 8 |
| Allocation concealment mechanism | 9 | Mechanism used to implement the random allocation sequence, describing any steps taken to conceal the sequence until interventions were assigned^§^ |  | p. 8 |
| Implementation | 10 | Who generated the random allocation sequence, who enrolled participants, and who assigned participants to interventions^§^ |  | p. 8 |
| Awareness of assignment | 11a | Who was aware of intervention assignment after allocation (for example, participants, providers, those assessing outcomes), and how any masking was done |  | p. 8 |
|  | 11b | If relevant, description of the similarity of interventions |  | N/A |
| Analytical  methods | 12a | Statistical methods used to compare group outcomes^§^ | How missing data were handled, with details of any imputation method | p. 12/13 |
|  | 12b | Methods for additional analyses, such as subgroup analyses, adjusted analyses, and process evaluations |  | p. 13-15 |
| **RESULTS** | | | | |
| Participant flow (a diagram is strongly recommended) | 13a | For each group, the numbers randomly assigned, receiving the intended intervention, and analysed for the outcomes^§^ | Where possible, the number approached, screened, and eligible prior to random assignment, with reasons for non-enrolment | Figure 1 |
|  | 13b | For each group, losses and exclusions after randomisation, together with reasons^§^ |  | Figure 1 |
| Recruitment | 14a | Dates defining the periods of recruitment and follow-up |  | p. 15 |
|  | 14b | Why the trial ended or was stopped |  | Click here to enter text. |
| Baseline data | 15 | A table showing baseline characteristics for each group^§^ | Include socioeconomic variables where applicable | Table 1 |
| Numbers analysed | 16 | For each group, number included in each analysis and whether the analysis was by original assigned groups^§^ |  | ITT; complete sample if not otherwise indicated |
| Outcomes and estimation | 17a | For each outcome, results for each group, and the estimated effect size and its precision (such as 95% confidence interval)^§^ | Indicate availability of trial data | Tables 2 to 5 |
|  | 17b | For binary outcomes, the presentation of both absolute and relative effect sizes is recommended |  |  |
| Ancillary analyses | 18 | Results of any other analyses performed, including subgroup analyses, adjusted analyses, and process evaluations, distinguishing pre-specified from exploratory |  | p. 1 – 20 |
| Harms | 19 | All important harms or unintended effects in each group (for specific guidance see CONSORT for Harms) |  | p. 18 |
| **DISCUSSION** | | | | |
| Limitations | 20 | Summarize the main results (including an overview of concepts, themes, and types of evidence available), link to the review questions and objectives, and consider the relevance to key groups. | Trial limitations, addressing sources of potential bias, imprecision, and, if relevant, multiplicity of analyses | p. 20 |
| Generalisability | 21 | Discuss the limitations of the scoping review process. | Generalisability (external validity, applicability) of the trial findings^§^ | p. 24/25 |
| Interpretation | 22 | Provide a general interpretation of the results with respect to the review questions and objectives, as well as potential implications and/or next steps. | Interpretation consistent with results, balancing benefits and harms, and considering other relevant evidence | p. 20 – 24 |
| **IMPORTANT INFORMATION** | | | | |
| Registration | 23 | Registration number and name of trial registry |  | p. 7 |
| Protocol | 24 | Where the full trial protocol can be accessed, if available |  | p. 7 |
| Declaration of Interests | 25 | Sources of funding and other support; role of funders | Declaration of any other potential interests | p. 27 |
| Stakeholder investments | 26a |  | Any involvement of the intervention developer in the design, conduct, analysis, or reporting of the trial | N/A |
|  | 26b |  | Other stakeholder involvement in trial design, conduct, or analyses | N/A |
|  | 26c |  | Incentives offered as part of the trial | N/A |

This table lists items from the CONSORT 2010 checklist (with some modifications for social and psychological intervention trials) and additional items in the CONSORT-SPI 2018 extension. Empty rows in the ‘CONSORT-SPI 2018’ column indicate that there is no extension to the CONSORT 2010 item

*We strongly recommended that the CONSORT-SPI 2018 Explanation and Elaboration (E&E) document be reviewed when using the CONSORT-SPI 2018 checklist for important clarifications on each item

§An extension item for cluster trials exists for this CONSORT 2010 item

This checklist is derived from:

- Montgomery, P., Grant, S., Mayo-Wilson, E., Macdonald, G., Michie, S., Hopewell, S., & Moher, D. (2018). Reporting randomised trials of social and psychological interventions: the CONSORT-SPI 2018 Extension. *Trials*, *19*(1), 407.
- Grant, S., Mayo-Wilson, E., Montgomery, P., Macdonald, G., Michie, S., Hopewell, S., & Moher, D. (2018). CONSORT-SPI 2018 Explanation and Elaboration: guidance for reporting social and psychological intervention trials. *Trials*, *19*(1), 406.
- Schulz, K. F., Altman, D. G., & Moher, D. (2010). CONSORT 2010 Statement: updated guidelines for reporting parallel group randomised trials. *BMJ*, *340*, c332.

Montgomery 2018 and Grant 2018 were distributed under the terms of the Creative Commons Attribution 4.0 International License ([http://creativecommons.org/licenses/by/4.0/](https://urldefense.com/v3/__http:/creativecommons.org/licenses/by/4.0/__;!!C5qS4YX3!CdcaroZO5hxp8FYacDXbUMpysBqFhPEDSGHr-PBP-6JeLAF0ufQ5lz_kopWiil164numaGA4JU3gaxY-WqOg1G1v39F-mLM$)). Schulz 2010 was distributed under the terms of a Creative Commons Attribution Non-commercial License (<https://creativecommons.org/licenses/by-nc/2.0/>). We have revised the checklists as published to include an extra column for “reporting on page #”.
